# Effect of unilateral renal ischemia on the contralateral kidney assessed by Caspase 3 expression

**DOI:** 10.1590/1677-5449.210040

**Published:** 2021-07-12

**Authors:** Carolina Rodrigues Dal Bo, Vitória Penido de Paula, Anna Paula Weinhardt Baptista Strazzi, Nelson Wolosker, Thiago Pinheiro Arrais Aloia, Angela Mazzeo, Oskar Grau Kaufmann

**Affiliations:** 1 Faculdade Israelita de Ciências da Saúde Albert Einstein – FICSAE, São Paulo, SP, Brasil.; 2 Hospital Israelita Albert Einstein – HIAE, São Paulo, SP, Brasil.; 3 Instituto de Ensino e Pesquisa – IIEP, Hospital Albert Einstein, São Paulo, SP, Brasil.; 4 Faculdade de Medicina da Universidade de São Paulo – USP, São Paulo, SP, Brasil.

**Keywords:** apoptosis, kidney, ischemia, immunohistochemistry, Caspase 3

## Abstract

**Background:**

Studies have demonstrated with histological analysis and Doppler flow measurement analysis that unilateral renal ischemia, which is performed in some surgeries, interfered with the contralateral kidney, identifying the phenomenon of kidney-kidney crosstalk.

**Objectives:**

To identify the effects on the ischemic and contralateral kidney of renal ischemia induced by two types of clamping technique by analyzing the volume of kidney cells positive for Caspase 3.

**Methods:**

Sixteen pigs were divided into 2 groups, as follows: A (n = 8) – clamping of left renal artery only and AV (n = 8) – clamping of left renal artery and vein. Immunohistochemical analyses (anti Caspase 3) were conducted with biopsy specimens collected from the ischemic and contralateral kidney at 0, 30, 60, and 90 minutes of ischemia and morphometric analysis was performed, taking the mean to represent the volume of the Caspase 3 positive area (%).

**Results:**

Morphometric analysis of specimens collected at 30, 60, and 90 minutes of ischemia showed that the mean area marked for Caspase 3 was statistically larger in the contralateral kidney than the ischemic kidney in both groups: clamped renal artery (A) and clamped renal artery and vein (AV). Comparing the ischemic and contralateral kidney, there was no statistically significant difference in the area marked for Caspase 3 between the two types of clamping.

**Conclusions:**

In the experimental model of unilateral renal ischemia, the non-ischemic kidney exhibited cell damage, demonstrated by Caspase 3 expression. The type of hilum clamping does not appear to influence the area marked for Caspase 3.

## INTRODUCTION

Temporary occlusion of renal blood flow by clamping the renal artery or simultaneously clamping the renal artery and vein is a procedure routinely employed in partial nephrectomy in order to reduce intraoperative bleeding.^1^-^4^ Patients who undergo this procedure may develop transitory or even definitive acute renal failure and the duration of clamping is a determinant factor.

The relationship between the risk of renal injury and the type of clamping performed is controversial in the literature – some studies suggest that simultaneously occluding both arterial and venous flow causes less renal damage after surgery. Other studies suggest that clamping the renal artery only is the better approach, claiming that the presence of venous flow causes less kidney damage.^5^,^6^

Several experimental studies^7^-^9^ have demonstrated that renal ischemia interferes to reduce the function of distant organs, such as the liver, heart, brain, and lungs, in a mechanism known as organ crosstalk. Two recent studies observed that unilateral renal ischemia affected the contralateral kidney, identifying the phenomenon of kidney-kidney crosstalk, demonstrated by histological analysis and by increases in resistance and pulsatility indexes calculated with Doppler flow measurement.^10^,^11^

Studies have shown that immunohistochemical analysis is a technique with high sensitivity for early detection of ischemic injury. Some authors suggest it can be used to plug gaps left by histological analysis of tissue ischemia.^12^-^15^ One immunohistochemical marker, Caspase 3, is a known marker of apoptosis that has been described in ischemic damage to several organs, including the brain, heart, and liver.^16^

We reviewed the literature and found no studies that have investigated the effect of unilateral renal ischemia on the contralateral kidney using immunohistochemical Caspase 3 analysis.

## OBJECTIVE

The objective of this study was to identify the effect of renal ischemia by clamping of the left renal artery only and by simultaneous clamping of the left renal artery and vein on the ischemic kidney and the contralateral kidney, analyzing the volume of renal cells positive for Caspase 3 over the course of the time in ischemia.

## METHODS

The surgical study was conducted between January and December of 2016 at the Surgical Experimentation and Training Center at the Hospital Israelita Albert Einstein (CETEC), accredited by the American Association for Assessment and Accreditation of Laboratory Animal Care (AAALAC), is in accordance with the standards set out by the National Board of Control for Animal Experimentation (CONCEA), and was approved by the Animal Use and Care Committee at the Hospital Israelita Albert Einstein (protocol CEUA 3290). The immunohistochemical analysis was performed at a laboratory run by the Instituto Israelita de Ensino e Pesquisa Albert Einstein (IIEP), from April 2018 to April 2019.

Sixteen Large White pigs, aged from 90 to 120 days, and weighing 30 kg each were divided into two groups of eight animals according to the type of renal clamping: group AV, with clamping of both the left renal artery and vein, and group A, with clamping of the left renal artery only. Biopsies were taken concomitantly from each animals’ ischemic kidney and contralateral non-ischemic kidney (the right kidney was the non-ischemic kidney) at 0, 30, 60, and 90 minutes of ischemia. Biopsies were processed in paraffin blocks and cut into 5 µm slices which were mounted on slides and used for immunohistochemical analysis.

### Anesthetic procedure and intraoperative technique

All of the animals were anesthetized in advance with intramuscular injection of Ketamine (10.0 mg/kg) and Midazolam (0.25 mg/kg), mixed in the same syringe. Fifteen minutes after the injection, the marginal vein of the ear was catheterized with a 20 or 22 caliber catheter (BD Insystem, BectonTherapy Systems Inc., Utah, United States)^17^ to provide venous access for anesthetic induction, which was accomplished using 7 mg/kg of Thiopental. Size 6.5 to 8.5 endotracheal tubes (Portex^®^, Minnesota, USA) were used to intubate the animals. Anesthesia was maintained with inhaled Isoflurane 2%. Analgesia was maintained with Fentanyl, at an initial dose of 2.5 µg/kg.^18^

With the animal anesthetized and in the dorsal decubitus position, the site was cleaned and surgical fields were positioned. All animals were subjected to invasive hemodynamic monitoring of arterial blood pressure (BP).

A midline xipho-pubic laparotomy was performed, with access to the retroperitoneal space, and the intestines were displaced. After dissection to reveal the renal vessels bilaterally, the diameter of the renal artery was measured and noted (mean diameter: 3-4 mm) and Doppler flow pattern was observed, at t=0, to rule out any preexisting abnormalities. After confirming that both kidneys were morphologically adequate for analysis, with maximum volume of 12 cm,^3^ the baseline biopsies were taken from both kidneys.

Next, the left renal hilum was clamped using a bulldog vascular clamp and the right kidney was left without ischemia. Serial biopsies were taken from the renal parenchyma, of both kidneys, at 30, 60, and 90 minutes.

Biopsy specimens were standardized at 1x1 cm^2^ from the cortical region. The serial biopsies did not negatively impact the kidneys, because they were taken from different sites in the cortex. After 90 minutes, the surgical clamps were removed and at the end of the surgical procedure the animals were euthanized under general anesthesia with an intravenous (IV) overdose of Thiopental and potassium chloride 19.1% (dose 15-30 mg/kg)

### Immunohistochemical analysis

For immunohistochemical analysis, the renal biopsy specimens collected at 0, 30, 60, and 90 minutes were fixed in formol 10% for 24 hours, dehydrated in a series of alcohol solutions of increasing concentrations, diaphanized in xylol, and set in paraffin. Four 5 µm thick slices mounted on silanized slides were used for each biopsy specimen/animal.

Once deparaffinized, the slides were hydrated and placed in a 10 mM (pH 6.0) solution of citric acid, which was heated for 12 minutes in a microwave oven for antigen recovery. Once cooled, the slides were incubated in a solution of 3% H_2_O_2_ to block endogenous peroxidase. Later, the slides were washed three times in phosphate buffered saline solution (PBS) with 0.5% Tween 20 soap for 5 minutes and incubated with a protein blocker (LowProteinBlocking, eBioscience, ThermoFisherScientific Inc., Massachusetts, United States). After being washed again in Tween 20, the slides were incubated overnight with rabbit polyclonal anti-Caspase 3 primary antibody (ThermoFisherScientific Inc., Massachusetts, United States).

On the following day, the slides were washed in Tween 20 and incubated with goat anti-IgG (H+L) secondary antibody (ThermoFisherScientific Inc., Massachusetts, United States). Slides were revealed with Diaminobenzidine-DAB (eBioscience ThermoFisherScientific Inc., Massachusetts, United States) and counterstained with hematoxylin for 1 minute.

Finally, the slides were dehydrated, immersed in xylol and protected with coverslips for analysis under an AxioVert A1 microscope (Zeiss, Germany) and microphotographed with an Axio CAM 503 Color digital camera (Zeiss, Germany). One slide was processed per animal, per time point (0, 30, 60, and 90 minutes), per kidney (ischemic and non-ischemic).

A morphometric analysis was conducted to calculate the volume of cells positive (%) for Caspase 3 using OLYMPUS cellSens Dimension software (Olympus Corporation, Tokyo, Japan). We took photos of eight slides per animal. Each slide had four slices from the same biopsy paraffin block, of which the first slice was the negative control for the reaction. Photos were taken of ten fields at random, with the mean result taken as the volume of Caspase 3 positive area per animal, expressed as a percentage.

### Statistical analysis

This experimental research study generated data in a controlled, prospective, and randomized manner. Data were subjected to statistical analysis using R (The R Foundation, Vienna, Austria).^19^ The measures analyzed are all quantitative and were expressed as means and standard deviations. The significance level was set at 5%.

Considering the low variability between animals and respecting the 3Rs bioethics principle for experimental studies with animals (replacement, reduction, and refinement) in defining the sample size, we conducted the study with 16 pigs, and, on the basis of the preliminary data, the variables were evaluated in terms of their distributions using quantile graphs, histograms, and box plots, in addition to the Shapiro-Wilk test. It was observed that the majority of data were concentrated close to the mean and the data varied little with relation to the mean. The assumption of normal distribution was not ruled out. This suggests that the sample represented the study population well and statistical tests could be applied with confidence, so it was unnecessary to subject additional animals to experimentation.

Comparison between the marked areas in groups differentiated by the type of clamping (A, unilateral renal artery, or AV, unilateral renal artery and vein) and kidney laterality (right, non-ischemic, or left, ischemic) were made using mixed linear regression models in order to account for the dependent nature of different measures from the same animal.^19^-^21^

## RESULTS

**Figure 1:**
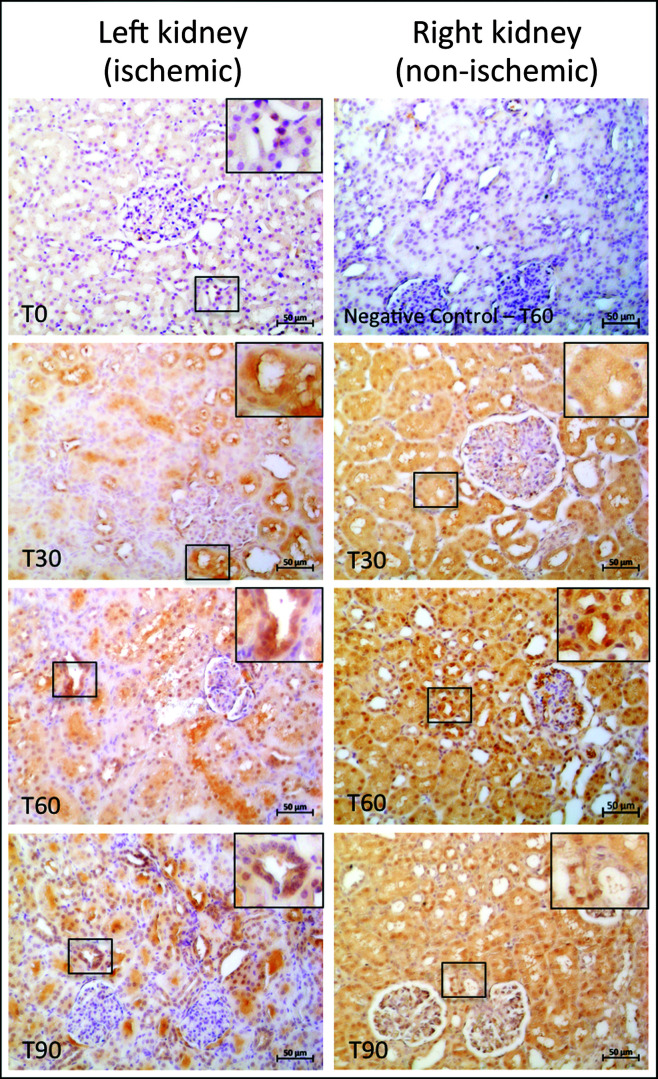
Immunostaining for Caspase 3 in convoluted renal tubules, indicated by the chestnut-brown color. Staining is cytoplasmic and nuclear, and the images compare the ischemic kidney (left kidney) to the non-ischemic kidney (right kidney) at 0, 30, 60, and 90 minutes of ischemia. (T0) Left kidney without clamp (positive control); (Negative control – T60) Negative reaction control; (T30) 30 minutes of unilateral renal ischemia; (T60) 60 minutes of unilateral renal ischemia; (T90) 90 minutes of unilateral renal ischemia; Bar: 50 μm.

The estimated mean area marked for Caspase 3 in the ischemic kidney and the contralateral kidney in group A (unilateral clamping of the renal artery) was not statistically different to the mean in group AV (unilateral clamping of the renal artery and vein) at any of the time points (Tables 1 and 2). In the group in which unilateral clamping of the renal artery and vein was performed (group AV), the mean estimated area marked for Caspase 3 in the non-ischemic kidney was not statistically greater than the mean area for the ischemic kidney at any of the time points, except for baseline (t=0) (Table 3 and Figure 1).

**Table 1:** Area marked for Caspase 3 in the ischemic kidney in groups A and AV and result of the mixed linear regression model for area marked as outcome.

**Duration of ischemia**	**A (%)**	**AV (%)**	**DM (95%CI)**	**p value**
**0 minutes**	0.02 (0.02)	0.02 (0.04)	0.00 (-0.02; 0.02)	0.953
**30 minutes**	0.24 (0.20)	0.20 (0.16)	-0.04 (-0.23; 0.15)	0.637
**60 minutes**	0.11 (0.13)	0.13 (0.12)	0.02 (-0.15; 0.18)	0.838
**90 minutes**	0.55 (0.36)	0.70 (0.34)	0.15 (-0.20; 0.51)	0.354

DM = difference in ischemic kidney means between groups A and AV; CI = confidence interval; p value according to the mixed linear regression model; Standard deviation in parentheses.

**Table 2:** Mean volume of area marked for Caspase 3 in the contralateral (non-ischemic) kidney in groups A and AV and result of the mixed linear regression model for area marked as outcome.

**Duration of ischemia**	**A (%)**	**AV (%)**	**DM (95%CI)**	**p value**
**0 minutes**	0.00 (0.00)	0.03 (0.05)	0.03 (-0.01; 0.06)	0.146
**30 minutes**	1.01 (0.35)	1.11 (0.50)	0.11 (-0.25; 0.46)	0.528
**60 minutes**	1.24 (0.79)	1.24 (0.71)	-0.01 (-0.68; 0.65)	0.965
**90 minutes**	0.84 (0.57)	1.11 (0.49)	0.29 (-0.21; 0.79)	0.229

DM = difference in contralateral kidney means (non-ischemic) between groups A and AV; CI = confidence interval; p value according to the mixed linear regression model; Standard deviation in parentheses.

**Table 3:** Mean volume of area marked for Caspase 3 in group AV in the ischemic kidney and the non-ischemic kidney and result of the mixed linear regression model for area marked as outcome.

**Duration of ischemia**	**Non-ischemic kidney (%)**	**Ischemic kidney (%)**	**DM (95%CI)**	**p value**
**0 minutes**	0.03 (0.05)	0.02 (0.04)	-0.01 (-0.02; 0.00)	0.135
**30 minutes**	1.11 (0.50)	0.20 (0.16)	-1.03 (-1.14; -0.91)	< 0.001
**60 minutes**	1.24 (0.71)	0.13 (0.12)	-1.15 (-1.33; -0.98)	< 0.001
**90 minutes**	1.11 (0.49)	0.70 (0.34)	-0.41 (-0.56; -0.26)	< 0.001

DM = difference between means for ischemic kidney and non-ischemic kidney in group AV; CI = confidence interval; p value according to the mixed linear regression model; Standard deviation in parentheses.

In the group in which unilateral clamping of the renal artery was performed (group A), the mean estimated area marked for Caspase 3 in the non-ischemic kidney was greater than the mean area for the ischemic kidney at all time points and this difference was statistically significant (Table 4).

**Table 4:** Mean volume of area marked for Caspase 3 in group A in the ischemic kidney and the non-ischemic kidney and result of the mixed linear regression model for area marked as outcome.

**Duration of ischemia**	**Non-ischemic kidney (%)**	**Ischemic kidney (%)**	**DM (95%CI)**	**p value**
**0 minutes**	0.00 (0.00)	0.02 (0.02)	0.02 (0.01; 0.02)	< 0.001
**30 minutes**	1.01 (0.35)	0.24 (0.20)	-0.79 (-0.90; -0.69)	< 0.001
**60 minutes**	1.24 (0.79)	0.11 (0.13)	-1.29 (-1.48; -1.11)	< 0.001
**90 minutes**	0.84 (0.57)	0.55 (0.36)	-0.27 (-0.42; -0.13)	< 0.001

DM = difference between means for ischemic kidney and non ischemic kidney in group A; CI = confidence interval; p value according to the mixed linear regression model; Standard deviation in parentheses.

## DISCUSSION

Among the animals subjected to clamping of the renal artery alone, there was a statistically significant difference at baseline between the ischemic kidney and the non-ischemic kidney. Despite this, as the ischemia time elapsed (30, 60, and 90 minutes), the non-ischemic kidney exhibited significant progression of the injury, which indicates that the consequences of renal ischemia are reflected in the non-ischemic kidney.

Among the animals subjected to simultaneous clamping of the renal artery and vein, there was no statistically significant difference at baseline in area marked for Caspase 3 between the ischemic kidney and the non-ischemic kidney. As the time elapsed, the area marked for Caspase 3 increased in the non-ischemic kidney, which could indicate that the cellular apoptosis in the ischemic kidney was also reflected in the contralateral kidney.

Ischemic kidney injury generally results in damage to cells of the nephron and cells of the renal vasculature. These cells are lost through a process of necrosis and apoptosis, which can lead to kidney failure.^22^ Ischemia caused by obstruction of the arteries of the kidney impairs the supply of nutrients and oxygen, changing cellular respiration and, consequently, renal metabolism through a cascade of biochemical reactions triggered by lack of energy (ATP).^23^ Additional damage is also observed, such as changes to the cellular cytoskeleton and polarity of surface membranes, activation of phospholipases and proteases and, finally, incapacity of cellular functions dependent on the ATPase enzyme (sodium-potassium pump).^24^

After the surgical clamping maneuver, blood flow is reestablished and, in this scenario, renal reperfusion intensifies the injuries caused by ischemia through production of reactive oxygen species, in addition to occurrence of cellular disarrangement, hypercoagulability, and congestion of microcirculation. This provokes reduction of renal blood flow, which characterizes the clinical status of renal failure.^25^ From this perspective, considering that in the present study the non-ischemic kidney underwent greater apoptosis than the ischemic kidney, one explanation could be that the ischemia is momentarily protecting the kidney that has had the renal hilum is clamped.

The phenomenon of organ crosstalk can be defined as mutual biological communication between distant organs mediated by signaling factors. This phenomenon helps to coordinate and maintain homeostasis, but a sudden dysfunction in any organ causes dysregulation in another organ.^26^

This interference appears to be linked to the inflammatory cascade generated by the ischemia mechanism. However, as far as we know, there are no experimental studies that prove that immunomarkers from the ischemic kidney interfere in the function of the contralateral kidney, that has not suffered ischemia.

A study that conducted histological analysis of pig kidneys to investigate the phenomenon of kidney-kidney crosstalk demonstrated a significant increase (p < 0.05) in the number of lesions to the control kidney after 10-20 minutes of ischemia induced by clamping the renal artery only, but only after 50-60 minutes of ischemia induced by clamping both renal artery and vein.^10^

However, with regard to apoptosis, the present study did not identify a statistically significant difference in the area marked for Caspase 3 between the group in which only the renal artery was clamped and the group in which both the artery and the vein were clamped.

With regard to limitations, this was an experimental study design using pigs because they are excellent biomedical models, given the anatomic, physiological, and immunological similarities to human beings. However, their kidneys have greater tolerance to ischemia duration than the human kidney.^27^

Unilateral renal ischemia was induced for 90 minutes in an attempt to identify any possible difference between the two types of clamping (renal artery only vs. renal artery and vein). After 120 minutes of surgery, on average, the majority of the animals were hypotensive and unresponsive as a result of dehydration, hypothermia and, possibly, intolerance of the anesthetic.

The difficulty in keeping the animals alive meant that our sample size was insufficient to assess the safety of the effect of renal reperfusion on the contralateral kidney, which should be considered in future investigations.

## CONCLUSIONS

In the experimental model of unilateral renal ischemia, the non-ischemic kidney suffered acute cellular damage, demonstrated by Caspase 3 expression, caused by the contralateral ischemia. The type of clamping of the hilum (unilateral renal artery vs. unilateral renal artery and vein) did not appear to have an effect on the volume of area marked for Caspase 3.
