# Supplementary material for: Effect of unilateral renal ischemia on the contralateral kidney assessed by Caspase 3 expression
Source: J Vasc Bras. 2021 Jul 12;20:e20210040. doi: 10.1590/1677-5449.210040 (PMC8294808; doi:10.1590/1677-5449.210040)
Supplement: Translation

# Translation: Efeito da isquemia renal unilateral no rim contralateral avaliada pela expressão de Caspase 3

**Authors:** Carolina Rodrigues Dal Bo, Vitória Penido de Paula, Anna Paula Weinhardt Baptista Strazzi, Nelson Wolosker, Thiago Pinheiro Arrais Aloia, Angela Mazzeo, Oskar Grau Kaufmann

**Affiliations:** 1 Faculdade Israelita de Ciências da Saúde Albert Einstein – FICSAE, São Paulo, SP, Brasil.; 2 Hospital Israelita Albert Einstein – HIAE, São Paulo, SP, Brasil.; 3 Instituto de Ensino e Pesquisa – IIEP, Hospital Albert Einstein, São Paulo, SP, Brasil.; 4 Faculdade de Medicina da Universidade de São Paulo – USP, São Paulo, SP, Brasil.

**Keywords:** apoptose, rim, isquemia, imuno-histoquímica, Caspase 3

## Abstract

**Contexto:**

Estudos demonstraram, por análise histológica e Dopplerfluxométrica, a interferência da isquemia renal unilateral, realizada em algumas cirurgias, sobre o rim contralateral, identificando o fenômeno de *kidney-kidney crosstalk*.

**Objetivos:**

Identificar o efeito da isquemia de duas estratégias de oclusão da vasculatura renal esquerda sobre o rim contralateral através do volume de células renais positivas para Caspase 3.

**Métodos:**

Suínos foram divididos em 2 grupos: A (n = 8), artéria renal esquerda clampeada, e AV (n = 8), artéria e veia renais esquerdas clampeadas. Foi realizado o estudo imuno-histoquímico (anti-Caspase 3), com o material de biópsias coletadas do rim isquêmico e contralateral em 0, 30, 60 e 90 minutos de isquemia, e análise morfométrica, sendo que a média representou o volume de área de Caspase 3 positiva (%).

**Resultados:**

A análise morfométrica do rim contralateral nos tempos 30, 60 e 90 minutos de isquemia mostrou que a média da área marcada por Caspase 3 foi estatisticamente superior à média do rim isquêmico nos dois grupos: artéria renal clampeada (A) e artéria e veia renais clampeadas (AV). Comparando o rim isquêmico e contralateral nos dois tipos de clampeamento, não houve diferença estatisticamente significante da área marcada por Caspase 3.

**Conclusões:**

No modelo experimental de isquemia renal unilateral, o rim não isquêmico apresentou dano celular, demonstrado pela expressão da Caspase 3 de forma aguda em decorrência da isquemia contralateral. O tipo de clampeamento do hilo não parece ter influência sobre o volume de área marcada por Caspase 3.

## INTRODUÇÃO

A interrupção temporária do fluxo sanguíneo renal por meio do clampeamento da artéria renal ou do clampeamento simultâneo da artéria e da veia renais é um procedimento adotado rotineiramente na nefrectomia parcial a fim de reduzir sangramento intraoperatório^1^^-^^4^. Os pacientes submetidos a esse procedimento podem desenvolver insuficiência renal aguda transitória e até definitiva, e o tempo de clampeamento é um fator determinante.

A relação entre o risco de lesão renal e o tipo de clampeamento realizado é controverso na literatura – alguns estudos indicam que a interrupção simultânea do fluxo arterial e venoso gera menos lesão renal após a cirurgia. Outros estudos sugerem que o clampeamento apenas da artéria renal seja uma melhor abordagem, justificando que a presença do fluxo venoso provoca menos danos ao rim^5^^,^^6^.

Diversos estudos experimentais^7^^-^^9^ demonstraram a interferência da isquemia renal na diminuição da função de órgãos à distância, como fígado, coração, cérebro e pulmão, em um mecanismo reconhecido como *organ crosstalk*. Dois estudos, recentemente, observaram a interferência da isquemia renal unilateral sobre o rim contralateral, identificando o fenômeno de *kidney-kidney crosstalk*, demonstrado por análise histológica e por aumento dos índices de resistência e pulsatilidade na análise Dopplerfluxométrica^10^^,^^11^.

A imuno-histoquímica foi demonstrada em estudos como uma técnica com alta sensibilidade para detectar precocemente lesões isquêmicas. Alguns autores sugerem o seu uso para complementar as lacunas da análise histológica na isquemia tecidual^12^^-^^15^. Entre os marcadores imuno-histoquímicos, a Caspase 3 é um marcador de apoptose conhecido e descrito em danos isquêmicos em vários órgãos, incluindo cérebro, coração e fígado^16^.

Após revisão da literatura, não identificamos estudos que avaliaram o efeito da isquemia renal unilateral sobre o rim contralateral através da análise imuno-histoquímica por Caspase 3.

## OBJETIVO

O objetivo deste estudo foi identificar o efeito da isquemia renal por clampeamento simples da artéria renal esquerda e do clampeamento simultâneo da artéria e da veia renal esquerdas sobre o rim isquêmico e sobre o contralateral, analisando o volume de células renais positivas para Caspase 3 ao longo do tempo de isquemia.

## MÉTODOS

O estudo operatório foi conduzido entre janeiro e dezembro de 2016 no Centro de Experimentação e Treinamento em Cirurgia do Hospital Israelita Albert Einstein (CETEC), acreditado pela Associação Americana para Acreditação de Cuidados com Animais de Laboratório (AAALAC), estando de acordo com as normas expedidas pelo Conselho Nacional de Controle da Experimentação Animal (CONCEA) e tendo sido aprovado pelo Comitê de Uso e Cuidado de Animais do Hospital Israelita Albert Einstein (protocolo CEUA 3290). A análise imuno-histoquímica foi realizada no laboratório do Instituto Israelita de Ensino e Pesquisa Albert Einstein (IIEP) entre abril de 2018 e abril de 2019.

Dezesseis suínos da raça Large White, com idade entre 90 e 120 dias e pesando 30 kg cada, foram divididos em dois grupos de acordo com o tipo de clampeamento renal, contendo oito animais cada: o grupo AV, com clampeamento de artéria e veia renais esquerdas, e o grupo A, com clampeamento da artéria renal esquerda. Os animais foram submetidos a biópsias no rim isquêmico e no rim contralateral (rim direito - não isquêmico) concomitantemente nos tempos 0, 30, 60 e 90 minutos de isquemia. As biópsias foram processadas em blocos de parafina e fragmentadas em cortes de 5 µm de espessura que, após serem fixados em lâminas, foram usados para análise imuno-histoquímica.

### Procedimento anestésico e técnica intraoperatória

Todos os animais foram previamente anestesiados com injeção intramuscular de Cetamina (10,0 mg/kg) e Midazolam (0,25 mg/kg), misturados na mesma seringa. Passados 15 minutos da injeção, a veia marginal da orelha foi cateterizada com cateter de calibre 20 ou 22 (BD Insystem, BectonTherapy Systems Inc., Utah, EUA)^17^, para servir de acesso venoso para a indução anestésica, que foi realizada com Tiopental 7 mg/kg. Para intubação dos animais, foram utilizados tubos endotraqueais tamanho 6,5 a 8,5 (Portex^®^, Minesota, EUA). A anestesia foi mantida inalatória com Isoflurano 2%. A analgesia foi mantida com Fentanil, com dose inicial de 2,5 µg/kg^18^.

Com o animal anestesiado na posição de decúbito dorsal horizontal, realizou-se a higienização local e colocação dos campos cirúrgicos. Todos os animais foram submetidos à monitorização hemodinâmica com pressão arterial (PA) invasiva.

Efetuou-se laparotomia xifopúbica mediana e acesso ao retroperitônio com mobilização das alças. Após dissecção e reparo dos vasos renais, bilateralmente, registrou-se a aferição do diâmetro da artéria renal (valor médio de 3-4 mm) e o padrão do fluxo renal ao Doppler, no tempo zero, para descartar qualquer alteração prévia. Após a certificação de que ambos os rins eram morfologicamente adequados para análise, com volume máximo de 12 cm^3^, efetuou-se biópsia no tempo zero, em ambos os rins.

Em seguida, foi realizado clampeamento do hilo renal esquerdo com *clamp* vascular tipo *bulldog*, e o direito permaneceu não isquêmico. A partir daí, foram realizadas biópsias seriadas do parênquima renal nos tempos 30, 60 e 90 minutos, em ambos os rins.

As biópsias foram padronizadas em 1x1 cm^2^ na região cortical. As biópsias seriadas não afetaram negativamente os rins, pois foram realizadas no córtex em diferentes locais. Após 90 minutos, as pinças cirúrgicas foram retiradas, e, ao final do procedimento cirúrgico, os animais foram eutanasiados sob anestesia geral com overdose de Tiopental e cloreto de potássio 19,1% intravenoso (IV) (dose 15-30 mg/kg)

### Análise imuno-histoquímica

Para a realização da análise imuno-histoquímica, as biópsias renais coletadas nos diferentes tempos foram fixadas em formol 10% por 24 horas, desidratados em soluções crescentes de álcool, diafanizados em xilol e incluídos em parafina. Foram utilizados quatro cortes de 5 µm de espessura realizados em lâminas silanizadas para cada fragmento da biópsia/animal.

Após desparafinizadas, as lâminas foram hidratadas e colocadas em solução de ácido cítrico 10 mM (pH 6,0), que foi aquecida por 12 minutos no micro-ondas para recuperação antigênica. Após resfriadas, as lâminas foram incubadas em solução de H_2_O_2_ 3% para bloqueio da peroxidase endógena. Posteriormente, as lâminas foram lavadas três vezes em solução salina tamponada (PBS) com sabão Tween 20 0,5% por 5 minutos e incubadas com um bloqueador de proteínas (LowProteinBlocking, eBioscience, ThermoFisherScientific Inc., Massachusetts, EUA). Após lavadas novamente em Tween 20, as lâminas foram incubadas *overnight* com anticorpo primário de coelho policlonal anti-Caspase 3 (ThermoFisherScientific Inc., Massachusetts, EUA).

No dia seguinte, as lâminas foram lavadas em Tween 20 e incubadas com anticorpo secundário de cabra anti-IGG (H+L) (ThermoFisherScientific Inc., Massachusetts, EUA). As lâminas foram reveladas por Diaminobenzidina-DAB (eBioscience ThermoFisherScientific Inc., Massachusetts, EUA) e contracoradas com hematoxilina por 1 minuto.

Finalmente, as lâminas foram desidratadas, imersas em xilol e protegidas por lamínulas para serem analisadas ao microscópio AxioVert A1 (Zeiss, Alemanha) e fotomicrografadas pela câmera digital Axio CAM 503 Color (Zeiss, Alemanha). Cada animal teve uma lâmina confeccionada por tempo (0, 30, 60 e 90 minutos) de cada rim (isquêmico e não isquêmico).

Foi realizada a análise morfométrica para calcular o volume de células positivas (%) para Caspase 3 utilizando o *software* OLYMPUS cellSens Dimension (OLYMPUS CORPORATION, Tóquio, Japão). Realizamos fotos de oito lâminas por animal. Cada lâmina continha quatro cortes do mesmo bloco parafinado da biópsia, sendo o primeiro corte o controle negativo da reação. Foram realizadas fotos de dez campos aleatórios, sendo que a média representou o volume de área de Caspase 3 positiva expressa em porcentagem por animal.

### Análise estatística

A realização desta pesquisa experimental gerou dados de forma controlada, prospectiva e randomizada. Os dados foram analisados estatisticamente usado o pacote estatístico R (The R Foundation, Viena, Áustria)^19^. As medidas avaliadas são todas quantitativas e foram descritas como médias e desvio padrão. O nível de significância adotado foi de 5%.

Considerando a variabilidade baixa entre animais e respeitando o princípio bioético dos 3Rs em estudo experimentais com animais (substituição, refinamento e redução), para definição do número amostral, realizamos um estudo com 16 suínos, e, a partir de dados preliminares, as variáveis foram avaliadas em relação à sua distribuição por meio dos gráficos de quantis, histograma e *boxplot*, além do teste de Shapiro-Wilk. Foi observado que a maior parte dos dados se concentrava próxima da média, e a variação dos dados em relação à média era pequena, não sendo rejeitada a suposição de normalidade. Isso sugere que a amostra representava bem a população estudada e era suficiente para aplicar testes estatísticos com segurança, não sendo necessário submeter mais animais ao estudo experimental.

A comparação entre as áreas marcadas nos grupos definidos a partir do tipo de clampeamento (A, artéria renal unilateral, e AV, artéria e veia renais unilaterais) e lado do rim (direito, não isquêmico, e esquerdo, isquêmico) foi feita por meio de modelos de regressão lineares mistos a fim de contemplar a dependência entre as diferentes medidas realizadas em um mesmo animal^19^^-^^21^.

## RESULTADOS

No rim isquêmico e no rim contralateral, a média estimada da área marcada por Caspase 3 no grupo A (clampeamento unilateral da artéria renal) não foi estatisticamente diferente da média do grupo AV (clampeamento unilateral da artéria e veia renais) em todos os momentos (Tabela1 e 2). No grupo em que foi realizado clampeamento unilateral da artéria e veia renal (grupo AV), a média estimada da área marcada por Caspase 3 no rim não isquêmico foi estatisticamente superior à média do rim isquêmico em todos os instantes de tempo, exceto no momento inicial (tempo zero) (Tabela 3 e Figura 1).

**Tabela 1:** Área marcada por Caspase 3 no rim isquêmico pelos grupos A e AV e resultado do modelo de regressão linear misto para a área marcada como desfecho.

**Tempo de isquemia**	**A (%)**	**AV (%)**	**DM (IC 95%)**	**Valor p**
**0 minuto**	0,02 (0,02)	0,02 (0,04)	0,00 (-0,02; 0,02)	0,953
**30 minutos**	0,24 (0,20)	0,20 (0,16)	-0,04 (-0,23; 0,15)	0,637
**60 minutos**	0,11 (0,13)	0,13 (0,12)	0,02 (-0,15; 0,18)	0,838
**90 minutos**	0,55 (0,36)	0,70 (0,34)	0,15 (-0,20; 0,51)	0,354

DM = diferença de médias entre os grupos A e AV no rim isquêmico; IC = intervalo de confiança; Valor-p referente ao modelo de regressão linear misto; Desvio padrão em parênteses.

**Tabela 2:** Média do volume de área marcada por Caspase 3 no rim contralateral (não isquêmico) pelos grupos A e AV e resultado do modelo de regressão linear misto para área marcada como desfecho.

**Tempo de isquemia**	**A (%)**	**AV (%)**	**DM (IC 95%)**	**Valor p**
**0 minuto**	0,00 (0,00)	0,03 (0,05)	0,03 (-0,01; 0,06)	0,146
**30 minutos**	1,01 (0,35)	1,11 (0,50)	0,11 (-0,25; 0,46)	0,528
**60 minutos**	1,24 (0,79)	1,24 (0,71)	-0,01 (-0,68; 0,65)	0,965
**90 minutos**	0,84 (0,57)	1,11 (0,49)	0,29 (-0,21; 0,79)	0,229

DM = diferença de médias entre os grupos A e AV no rim não isquêmico; IC = intervalo de confiança; Valor-p referente ao modelo de regressão linear misto; Desvio padrão em parênteses.

**Tabela 3:** Média do volume de área marcada por Caspase 3 no grupo AV no rim isquêmico e no não isquêmico e resultado do modelo de regressão linear misto para área marcada como desfecho.

**Tempo de isquemia**	**Rim não isquêmico (%)**	**Rim isquêmico (%)**	**DM (IC 95%)**	**Valor p**
**0 minuto**	0,03 (0,05)	0,02 (0,04)	-0,01 (-0,02; 0,00)	0,135
**30 minutos**	1,11 (0,50)	0,20 (0,16)	-1,03 (-1,14; -0,91)	< 0,001
**60 minutos**	1,24 (0,71)	0,13 (0,12)	-1,15 (-1,33; -0,98)	< 0,001
**90 minutos**	1,11 (0,49)	0,70 (0,34)	-0,41 (-0,56; -0,26)	< 0,001

DM = diferença de médias entre o rim isquêmico e não isquêmico no grupo AV; IC = intervalo de confiança; Valor-p referente ao modelo de regressão linear misto; Desvio padrão em parênteses.

**Figura 1:** Imunomarcação por Caspase 3 nos túbulos contorcidos renais representados pela cor marrom-acastanhado. A marcação é citoplasmática e nuclear, e as imagens comparam o rim isquêmico (rim esquerdo) com o rim não isquêmico (rim direito) nos tempos 0, 30, 60 e 90 de isquemia. (T0) Rim esquerdo sem *clamp* (controle positivo); (Negative control – T60) Controle negativo da reação; (T30) 30 minutos de isquemia renal unilateral; (T60) 60 minutos de isquemia renal unilateral; (T90) 90 minutos de isquemia renal unilateral; Barra: 50 μm.

No grupo em que foi realizado clampeamento unilateral da artéria renal (grupo A), a média estimada da área marcada por Caspase 3 do rim não isquêmico foi maior que a média do rim isquêmico em todos os instantes de tempo, sendo essa diferença estatisticamente significante (Tabela 4).

**Tabela 4:** Média do volume de área marcada por Caspase 3 no grupo A no rim isquêmico e no não isquêmico e resultado do modelo de regressão linear misto para área marcada como desfecho.

**Tempo de isquemia**	**Rim não isquêmico (%)**	**Rim isquêmico (%)**	**DM (IC 95%)**	**Valor p**
**0 minuto**	0,00 (0,00)	0,02 (0,02)	0,02 (0,01; 0,02)	< 0,001
**30 minutos**	1,01 (0,35)	0,24 (0,20)	-0,79 (-0,90; -0,69)	< 0,001
**60 minutos**	1,24 (0,79)	0,11 (0,13)	-1,29 (-1,48; -1,11)	< 0,001
**90 minutos**	0,84 (0,57)	0,55 (0,36)	-0,27 (-0,42; -0,13)	< 0,001

DM = diferença de médias entre o rim isquêmico e não isquêmico no grupo A; IC = intervalo de confiança; Valor-p referente ao modelo de regressão linear misto; Desvio padrão em parênteses.

## DISCUSSÃO

Nos animais submetidos ao clampeamento apenas da artéria renal, houve diferença estatisticamente significante no tempo zero entre o rim isquêmico e o não isquêmico. Apesar disso, ao longo do tempo (30, 60 e 90 minutos), o rim não isquêmico apresentou uma progressão de lesão significativa, o que indica que as consequências da isquemia renal são refletidas no rim não isquêmico.

Nos animais submetidos ao clampeamento simultâneo da artéria e da veia renal, no tempo inicial não havia diferença estatisticamente significante na área marcada por Caspase 3 entre o rim isquêmico e o rim não isquêmico. Com o passar do tempo, observou-se aumento da área marcada por Caspase 3 no rim não isquêmico, o que pode indicar que a apoptose celular sofrida pelo rim isquêmico também seja refletida no rim contralateral.

A lesão isquêmica do rim geralmente resulta em danos às células do néfron e às células da vasculatura renal. Essas células são perdidas através do processo de necrose e apoptose, podendo levar à falência renal^22^. A isquemia causada por uma obstrução nas artérias do rim prejudica a chegada de nutrientes e de oxigênio, alterando a respiração celular e, consequentemente, o metabolismo renal através de uma cascata de reações bioquímicas decorrentes da falta de energia (ATP)^23^. Outros danos também são verificados, tais como alteração no citoesqueleto celular, na polaridade da superfície das membranas, na ativação de fosfolipases e proteases e, por fim, na incapacidade das funções celulares dependentes da enzima ATPase (bomba de sódio-potássio)^24^.

Após a finalização da manobra cirúrgica de clampeamento, o fluxo sanguíneo é restabelecido, e a reperfusão renal, nesse cenário, intensifica as lesões causadas pela isquemia através da produção de espécies reativas de oxigênio, além da ocorrência de desarranjo celular, hipercoagulabilidade e congestão da microcirculação. Isso favorece a redução do fluxo sanguíneo renal, o que caracteriza o quadro clínico da insuficiência renal^25^. Nesse sentido, levando em consideração que, neste estudo, o rim não isquêmico sofreu maior apoptose que o rim isquêmico, uma explicação seria que a isquemia pode estar momentaneamente protegendo o rim em que o hilo renal está clampeado.

O fenômeno *organ crosstalk* pode ser definido como a comunicação biológica mútua entre órgãos distantes mediada por fatores de sinalização. Esse fenômeno ajuda a coordenar e manter a homeostase, mas uma súbita disfunção em qualquer órgão causa desregulação em outro órgão^26^.

Essa interferência parece estar envolvida com a cascata inflamatória gerada pelo mecanismo de isquemia. Entretanto, até onde sabemos, não há estudos experimentais que comprovem a interferência com imunomarcadores do rim isquêmico na função do rim contralateral, não isquêmico.

Um estudo que realizou análise histológica de rins de suínos para estudar o fenômeno *kidney-kidney crosstalk* demonstrou aumento significativo (p < 0,05) do número de lesões no rim controle a partir de 10-20 minutos de isquemia clampeando apenas a artéria renal e somente com 50-60 minutos de isquemia clampeando artéria e veia renais^10^.

No entanto, em relação à apoptose, o presente estudo não identificou diferença estatisticamente significante na área marcada por Caspase 3 entre o grupo em que foi clampeada a artéria renal exclusivamente e o grupo em que foi clampeada tanto a artéria quanto a veia renais.

Em relação às limitações, este estudo foi experimental, delineado com suínos, pois eles são excelentes modelos biomédicos dadas as semelhanças anatômicas, fisiológicas e imunológicas ao ser humano. Entretanto, os rins desses animais possuem uma maior tolerância ao tempo de isquemia do que o rim humano^27^.

A isquemia renal unilateral foi realizada até 90 minutos em uma tentativa de identificar qualquer diferença possível entre os dois tipos de clampeamento (apenas artéria renal vs. artéria e veia renais). Após 120 minutos de cirurgia, em média, a maioria dos animais encontrava-se em estado hipotensivo e sem resposta em decorrência de desidratação, hipotermia e, possivelmente, intolerância anestésica.

Devido à dificuldade em manter os animais vivos, tivemos uma amostra insuficiente para avaliação da segurança do efeito da reperfusão renal no rim contralateral, a qual deve ser considerada em investigações futuras.

## CONCLUSÃO

No modelo experimental de isquemia renal unilateral, o rim não isquêmico apresentou dano celular, demonstrado pela expressão da Caspase 3, de forma aguda em decorrência da isquemia contralateral. O tipo de clampeamento do hilo (artéria renal unilateral vs. artéria e veia renais unilaterais) não parece ter influência sobre o volume de área marcada por Caspase 3.
